# Genome-wide prediction of discrete traits using bayesian regressions and machine learning

**DOI:** 10.1186/1297-9686-43-7

**Published:** 2011-02-17

**Authors:** Oscar González-Recio, Selma Forni

**Affiliations:** 1 INIA. Ctra La Coruña km 7.5, 28040 Madrid. Spain; 2 Genus Plc, 100 Bluegrass Commons Blvd. Ste 2200. Hendersonville, TN, USA

## Abstract

**Background:**

Genomic selection has gained much attention and the main goal is to increase the predictive accuracy and the genetic gain in livestock using dense marker information. Most methods dealing with the large *p *(number of covariates) small *n *(number of observations) problem have dealt only with continuous traits, but there are many important traits in livestock that are recorded in a discrete fashion (e.g. pregnancy outcome, disease resistance). It is necessary to evaluate alternatives to analyze discrete traits in a genome-wide prediction context.

**Methods:**

This study shows two threshold versions of Bayesian regressions (Bayes A and Bayesian LASSO) and two machine learning algorithms (boosting and random forest) to analyze discrete traits in a genome-wide prediction context. These methods were evaluated using simulated and field data to predict yet-to-be observed records. Performances were compared based on the models' predictive ability.

**Results:**

The simulation showed that machine learning had some advantages over Bayesian regressions when a small number of QTL regulated the trait under pure additivity. However, differences were small and disappeared with a large number of QTL. Bayesian threshold LASSO and boosting achieved the highest accuracies, whereas Random Forest presented the highest classification performance. Random Forest was the most consistent method in detecting resistant and susceptible animals, phi correlation was up to 81% greater than Bayesian regressions. Random Forest outperformed other methods in correctly classifying resistant and susceptible animals in the two pure swine lines evaluated. Boosting and Bayes A were more accurate with crossbred data.

**Conclusions:**

The results of this study suggest that the best method for genome-wide prediction may depend on the genetic basis of the population analyzed. All methods were less accurate at correctly classifying intermediate animals than extreme animals. Among the different alternatives proposed to analyze discrete traits, machine-learning showed some advantages over Bayesian regressions. Boosting with a pseudo Huber loss function showed high accuracy, whereas Random Forest produced more consistent results and an interesting predictive ability. Nonetheless, the best method may be case-dependent and a initial evaluation of different methods is recommended to deal with a particular problem.

## Background

The availability of thousands of markers from high throughput genotyping platforms offers an exciting prospect to predict the outcome of complex traits in animal breeding using genomic information (the so-called genomic selection) and in personalized medicine. Besides production and other functional traits, genomic selection offers a novel challenge for discovering genetic variants affecting important diseases in humans, plants and livestock, and also for breeding resistant individuals to improve farm profitability.

The statistical treatment of the genetic basis of these traits is not straightforward because multiple genes, gene by gene interactions and gene by environment interactions underlie most complex traits and diseases. Capturing all marker signals is currently challenging. Besides the large *p *small *n *problem, the statistical treatment of the categorical nature of a trait may increase parameterization. So far, methods dealing with genome-assisted evaluations have focused on traits expressed or recorded in a continuous and Gaussian manner [1-3]. However, other traits (e.g. disease, survival) are generally recorded in a binary or few-classed manner (e.g. healthy or sick, number of occurrences, status). Most methods dealing with genome-assisted evaluations may be extended in a relatively well known manner to analyze categorical traits [4-6]. A larger amount and various types of genomic information (e.g. single nucleotide polymorphisms, copy number variants or DNA sequencing) for several species are likely to be available in the future. Using this large amount of data may be highly informative, yet quite challenging for current methods from the point of view of computation efficiency. Genome-wide association studies (GWAS) and genomic selection methods must be adapted to cope with these challenges.

Machine-learning is becoming more and more popular to deal with the difficulties stated above, and has been previously applied in GWAS in humans [7] and livestock [8-10]. Machine-learning methods aim at improving a predictive performance measure by repeated observation of experiences. They are model specification free, and may capture hidden information from large databases. This is appealing in a genomic information context in which multiple and complex relationships between genes exist. The ensemble methods, such as Random Forest (RF) algorithms [11] and boosting [12], are the most appealing alternatives to analyze complex discrete traits using dense genomic markers information, and have been previously applied in GWAS for human diseases [13,14]. They may provide a measurement of the importance of each marker on a given trait and good predictive performance. Boosting has been previously applied in a genomic selection context for regression problems using the *L_2 _*loss function [8]. RF and boosting do not require specification of the mode of inheritance and hence may account for non-additive effects. Further, they are fast algorithms, even when handling a large amount of covariates and interactions, and can be applied to both classification and regression problems.

The objective of this study was to present the threshold extension of two Bayesian regression methods that are used in genome-assisted evaluations (Bayes A and Bayesian LASSO), a boosting algorithm for discrete traits, to describe more thoroughly the RF alternative to deal with discrete traits in a genome-wide prediction context, and to apply them to both simulated and real data to compare their predictive ability.

## Methods

Let **y **={*y_i_*} be a vector of phenotypes recorded in a binary fashion (0/1) from *n *animals genotyped for *p *markers **X **= {**x***_i_*}. Four different methods were applied: two linear regressions using a Bayesian framework, and two machine-learning ensemble algorithms.

### Model 1: threshold Bayes A

A threshold version of Bayes A (TBA) model was proposed here, which is an extension of the Bayesian regression proposed by Meuwissen et al. [1]. The traditional threshold model [4] postulates that there is an underlying random variable, called liability (λ) that follows a continuous distribution, and that the observed dichotomy is the result of the position of the liability with respect to a fixed threshold (*t*):

$$\text{phenotype}=\left\{ \begin{array}{l} 0\;if\;\lambda <t\\ 1\;if\;\lambda \geq t \end{array}\right.$$

The liability is taken as the response variable. The proposed modification consists of the linear regression of the single nucleotide polymorphism (SNP) coefficients on a liability variable with Gaussian distribution. The TBA can be described as follows:

λ=μ1+Xb+e

where, **λ **is the underlying liability variable vector for **y**, *μ *is the population mean, **1 **is a column vector (*n*×1) of ones; **b **= {*b_j_*} corresponds to the vector for the regression coefficient estimates of the *p *markers or SNP assumed normally and independently distributed a priori as $N(0,\;\sigma_{j}^{2})$, where $\sigma_{j}^{2}$ is an unknown variance associated with marker *j*. The prior distribution of $\sigma_{j}^{2}$ is assumed to be distributed as the scaled inverse chi-square $\sigma_{j}^{2}\sim \upsilon_{j}s_{j}^{2}\chi_{\upsilon_{j}}^{-1}$, with *υ_j _*= 4 and $s_{j}^{2}=0.002$. Elements of the incidence matrix **X**, of order *n *× *p*, may be set up as for different additive, dominant or epistatic models. In the more practical scenario, it takes values -1, 0 or 1 for marker genotypes *aa*, *Aa *and *AA*, respectively. The residuals (**e**) are assumed to be distributed as $N(0,\;\sigma_{e}^{2})$, with residual variance $\sigma_{e}^{2}=1$, as stated above. As in a regular threshold model, two parameters have to be set fixed (e.g. threshold and the residual variance are set to zero and one, respectively) since these parameters are not identifiable in a liability model.

This method can be solved via the Gibbs sampler described in Meuwissen et al. [1], with the simple incorporation of the data augmentation algorithm to sample the individual liabilities from their corresponding truncated normal distribution as described in Tanner and Wong [15]. The joint posterior distribution of the *n *liabilities is:

$$\begin{array}{l} \text{Prob}\left(\lambda |\mu ,b,t\right)=\\ \prod_{i=1}^{n}\{\frac{\Phi [t-(\mu +x_{i}b)]}{\sigma_{e}}\}1-y_{i}{\left\{1-\frac{\Phi [t-(\mu +x_{i}b)]}{\sigma_{e}}\right\}}^{y_{i}} \end{array}$$

### Model 2: threshold Bayesian LASSO

The Bayesian LASSO described by Park and Casella [16] and its version for genomic selection detailed in de los Campos et al. [17] can also be extended to discrete traits [18]. As stated in the previous model, the response variable is a liability response (λ) that follows a continuous distribution. The Bayesian threshold LASSO (BTL) can be solved as:

λ=μ1+Xβ+e,

where **λ **is the vector of liabilities for all individuals, *μ *is the population mean, **1 **is a column vector (*n *× 1) of ones; $\overset{\wedge }{\beta }$ are the LASSO estimates with their respective incidence matrix **X **as described for model TBA. As a modeling choice, **e **was considered the vector of independently and identically distributed residuals, as $e∼N(0,\sigma_{e}^{2})$. In accordance with tradition, we fixed the threshold to be 0 and the residual variance to be 1 as described for model TBA; alternate choices result in the same model.

In a fully Bayesian context, the LASSO estimates $\overset{\wedge }{(\beta })$ can be interpreted as posterior modes estimates when the regression parameters have independent and identical double-exponential priors [19]. Park and Casella [16] have proposed a conditional Laplace prior specification for the LASSO estimates of the form:

$$p\left(\beta |\sigma_{e}^{2}\right)=\prod_{j=1}^{p}\frac{\gamma }{2\sqrt{\sigma_{e}^{2}}}e^{-\gamma |\beta_{j}|/\sqrt{\sigma_{e}^{2}}},$$

where $\sigma_{e}^{2}$ is the residual variance, and *γ *is a parameter controlling the shrinkage of the distribution. Inferences about *γ *may be done in different ways [16]. To follow the Bayesian specifications, a gamma prior is proposed here for *γ^2^*, with known rate (*r*) and shape (*δ*) hyper-parameters, as described by de los Campos et al. [17]. Samples from posterior distributions of those estimates may be drawn from the Gibbs sampling algorithm described in de los Campos et al. [17], with the corresponding data augmentation algorithm for liabilities, as described for TBA.

### Model 3: gradient boosting

Gradient boosting may be classified as an ensemble method [20]. This algorithm combines different predictors in a sequential manner with some shrinkage on them [12] and performs variable selection. Gradient boosting forms a "committee" of predictors with potentially greater predictive ability than that of any of the individual predictors in the form:

$$y=\mu +\sum_{m=1}^{M}vh_{m}(y;X)$$

Each predictor **(***h_m_***(y; X) **for *m *∈ (1, *M*)) is applied consecutively to the residual from the committee formed by the previous ones. This algorithm can be calculated using importance sampling learning ensembles as follows:

(Initialization): Given data **(y, X)**, let the prediction of phenotypes be *F*_0 _= *μ*, with *μ *being the population mean.

Then, for *m *in {1 to *M*}, with *M *being large, calculate the loss function (*L*) for $\left(y_{i},F_{m-1}(x_{i})+h(y_{i};x_{i},j_{m})\right)$

where *j_m _*is the SNP (only one SNP is selected at each iteration) that minimizes $\sum_{i=1}^{n}L\left(y_{i},F_{m-1}(x_{i})+h(y_{i};x_{i},j_{m})\right)$ at iteration *m*, *h*(*y_i_*; **x***_i_*, *j_m_*) is the prediction of the observation using SNP *j *at the current iteration, *F_m_*_-1_(**x***_i_*) is the updated prediction at the previous iteration and *L*(·) is a given loss function. The updated prediction at each iteration *m *may be expressed as *F_m_*(**x***_i_*) = *F_m_*_-1_(**x***_i_*)+*v*·*h*(*y_i_*; **x***_i_*, *j_m_*) with *v* being some shrinkage factor that, without loss of generality, can be assumed constant and small (0<*v* <1), but it may be optimized to balance predictive ability and computation time.

Therefore, after the initialization, the algorithm flows as follows:

Step 1: Compute residuals as $r_{m}=y-\sum_{i=0}^{m-1}v\cdot F_{m-1}(x_{i})$, and fit the weak learner for each SNP *j *(*j *∈{1,..., *p*}) to current residuals, where *ν *was set to 0.01.

Step 2: Select SNP *j*, where $j=\arg\min_{j}\sum_{i=1}^{n}L\left(y_{i},F_{m-1}(x_{i})+h(y_{i};x_{i},j_{m})\right)$, i.e. the SNP minimizing the loss function.

Step 3. Update predictions as *F_m_*(**x***_i_*) = *F_m_*_-1_(**x***_i_*)+*ν·h*(*y_i_*; **x***_i_*, *j_m_*), (*i*∈{1,..., *n*}), where *h*(*y_i_*; **x***_i_*, *j_m_*) is the estimate for individual *i *obtained by regressing the current residual (*r_i_*) at iteration *m *on its genotype for the SNP selected in step 2.

Step 4: Increase the iteration index *m *by 1, and repeat steps 2-4 until a convergence criterion is reached.

Here, we used ordinary least square regression as predictor *h*(**y**; **X**) and two different loss functions: the *L_2 _*loss function (L2B), which is a quadratic error term in the form (*y_i_*-*F*_*m *_(*y_i_*; **x***_i_*, *j_m_*))^2^, and a pseudo-Huber loss function (LhB) in the form $\log\left[\cosh\left(y_{i}-F_{m}(y_{i};x_{i},j_{m})\right)\right]$. The pseudo Huber loss function is a priori more appealing for discrete traits because it is continuous, differentiable, greater than or equal to the logit loss function and overcomes the disadvantage of the squared loss by becoming more linear when (*y_i_*-*F_m_*(*y_i_*; **x***_i_*, *j_m_*)) tends to infinite. The choice of the number of iterations, *M*, is a model comparison problem which may be overcome in many different ways [12,20]. Here, a cross-validation design was used as described in González-Recio et al. [8]. More details on the gradient boosting can be found in Freund and Schaphire [21], Friedman [12] and González-Recio et al. [8].

### Model 4: Random Forest

Random Forest can be viewed as a machine learning ensemble algorithm and was first proposed by Breiman [11]. It is massively non-parametric, robust to over-fitting and able to capture complex interaction structures in the data, which may alleviate the problems of analyzing genome-wide data. This algorithm constructs many decision trees on bootstrapped samples of the data set, averaging each estimate to make final predictions. This strategy, called bagging [22], reduces error prediction by a factor of the number of trees.

A RF algorithm aimed at genome-wide prediction is described next, in a more extensive manner than the previous methods, as this is the first time that this algorithm is used in a genomic breeding value prediction context:

Let **y **(*n *× 1) be the data vector consisting of discrete observations for the outcome of a given trait, and **X **= {**x***_i_*} where **x***_i _*is a (*p *× 1) vector representing the genotype of each animal (0, 1 or 2) for *p *SNP, to which *T *decision trees are built (see classification and regression tree theory e.g. [20]). Note that main SNP effects, SNP interactions, environmental factors or combinations thereof may be also included in **x***_i_*. This ensemble can be described as an additive expansion of the form:

$$y=\mu +\sum_{t=1}^{T}c_{t}h_{t}(y;X)$$

Each tree (*h_t_*(**y**; **X**) for *t*∈(1, *T*)) is distinct from any other in the ensemble as it is constructed from *n *samples from the original data set selected at random with replacement, and at each node only a small group of SNP are randomly selected to create the splitting rule. Each tree is grown to the largest extent possible until all the terminal nodes are maximally homogeneous. Then, *c*_t _is some shrinkage factor averaging the trees. The trees are independent identically distributed random vectors, each of them casting a unit vote for the most popular outcome of the disease at a given combination of SNP genotypes.

Each tree minimizes the average loss function of the bootstrapped data, and is constructed using a heuristic approach as follows:

1. First, bootstrapped samples from the whole data set are drawn with replacement so that realization (*y_i_*, **x**_*i*_) may appear several times or not at all in the bootstrapped set Ψ^(*t*) ^*t *= (*1*,..., *T*).

2. Then, draw *mtry *out of *p *SNP markers at random, and select the SNP *j*, *j*∈(1,..., *mtry*), where

$$j=\arg\min_{j}L(y,h_{t}(X)),$$

with *L*(*y*, *h_t_*(**X**)) being a certain loss function. i.e. SNP *j *is the one that minimizes a given loss function at the current node, and is selected in this step. The algorithm takes a fresh look at the data that have arrived at each node and evaluate all possible splits. Many loss functions can be chosen (e.g. logit function, squared loss function, misclassification rate, entropy, Gini index, ...). The behavior of a given loss function may depend on the nature of the problem. The squared loss function is popular for continuous response variables, and the logit function for categorical responses.

3. Split the node in two child nodes according to SNP *j *genotype that one individual may or may not have (e.g. individuals with the risk allele will pass to a child node, and the remaining animals will pass to the other child node).

4. Repeat steps 2-3 until a minimum node size is reached (usually <5). The predicted value of the genotype **x***_i _*is the majority vote for the outcome at the terminal nodes (for regression problems, it is the average phenotype of the individuals in the node).

Finally, a large amount of trees are constructed repeating steps 1-4 to grow a random forest. The forest may be stopped when the generalization error averaged across the out of bag samples (see section below) have converged. Convergence may be visually tested but it may also be determined using traditional methods for convergence testing of Monte Carlo Markov chains.

Final predictions can be made by averaging the values predicted at each tree to obtain a probability of being susceptible. In a naïve 0 = non-susceptible/1 = susceptible scenario, individuals with probability <0.5 may be considered as non-susceptible. To predict observations of new individuals, their marker genotypes are passed down each tree, and the estimate of the corresponding terminal nodes is assigned to the new individual in each tree. The predictions of each tree in the RF algorithm are averaged for each animal to compute the final prediction.

There are two main aspects that can be tuned in random forest: the first one is the number of SNP or covariates sampled at random for each node (*mtry*). Generalized cross-validation strategies can be used to optimize *mtry*. In high dimensional problems such as GWAS, Goldstein et al. [23] have suggested *mtry *to be fixed to >0.1 *p*. The algorithm may speed up for smaller *mtry *values. Nonetheless, cross-validation can be used to determine the best value of *mtry *for each trait, although at an expense of increasing computation time. Genetic background may influence the behavior of this tuning parameter. The second aspect is the criterion to select the best SNP to split the node. As commented above, different criteria may be used and the best choice may depend on the nature of the problem. Entropy theory seems the most appealing to evaluate genomic information on discrete traits (as concluded from pilot studies, results not shown). Other loss functions such as the *L*_1_-loss function or the misclassification rate could be implemented in an easy manner. Without loss of generality we show how to implement the entropy theory in the node splitting decision. The information gain (IG) for each covariate *s *drawn at random in a given node was calculated as described in Long et al. [9]:

Suppose there are $N_{k}^{+}$ individuals with genotype *k *(*k *∈ {0, 1, 2}) at each SNP covariate *x_j _*showing y = 1 (e.g. presence of disease) at such node, and $N_{k}^{-}$ individuals with the same genotype with y = 0 (e.g. absence of disease). The information gain for each covariate *x_j _*can be calculated as:

$$\begin{array}{c} IG(x_{j})=H(\mathrm{Pr}(Y))-\\ \sum_{k=1}^{2}\left(\frac{\sum_{C=+,-}N_{k}^{C}}{N}\left(-\sum_{C=+,-}\frac{N_{k}^{C}}{N_{k}}\log_{2}\frac{N_{k}^{C}}{N_{k}}\right)\right) \end{array}$$

where $N_{k}=N_{k}^{+}+N_{k}^{-}$, and $H(\mathrm{Pr}(y))=-\sum_{y\in A}\mathrm{Pr}(y)\log_{2}\mathrm{Pr}(y)$ is the entropy of the probability distribution of **y**, and *A *is the set of all states that **y **can take ({0,1}). The SNP covariate with the highest IG at each node is used to split the node into two new child nodes, each one containing the individuals from the parent node with the risk or the non-risk allele, respectively.

There are two features involved in the RF algorithm that deserve further attention: the out of bag samples, and the variable importance.

#### Out of bag sample

The out of bag data (OOB) is an interesting feature of RF. Each tree is grown using a bootstrapped sample of the data, which leaves roughly one third of the observations out because some animals will appear more than once and others will not appear at all. The samples that do not appear are called the OOB samples. The OOB acts as a tuning/validation set at each tree and is almost identical to a *n*-fold cross validation, removing the need for a set aside test or tune test. Tuning of parameters can be done along the RF using the OOB, and generalization error can be calculated as the error rate of the OOB [11,24].

#### Variable importance

RF may use the OOB to provide an importance measure of predictor variables (SNP or environmental effects). The relative variable importance (VI) is estimated as follows. After each tree is constructed, the OOB are passed down the tree and the prediction accuracy of disease outcome is calculated using the chosen criterion (e.g. misclassification rate, *L*_2 _loss function). Then, genotypes for the *p*th SNP are permuted in the OOB, and the accuracy for the permuted SNP is again calculated. The relative importance is calculated as the difference between these prediction accuracies (that of the original OOB and that of the OOB with the permuted variable). This step is repeated for each covariate (SNP) and the decrease of accuracy is averaged over all trees in the random forest. The variable importance may be expressed as a percentage of the accuracy obtained with the most important SNP, and provides insight in the level of association of the SNP with the disease. The SNP with higher VI may be of interest for prediction of trait susceptibility (e.g. disease resistance, low fertility) at low marker density, candidate gene studies or gene expression studies.

Our own java code has been developed for implementing RF for categorical or continuous traits under a genome-wide prediction context, and is available upon request to the authors.

### Data sets

Simulated and field data sets were used for the model comparisons. Description of these data is given next.

#### Simulated set

QMSim software [25] was run to simulate a population of thousands of animals genotyped for roughly 10,000 markers. First, 1000 historical generations were generated in a population with effective size decreasing from 1400 to 400 to mimic a bottleneck, in order to produce a realistic level of LD for the platform used in the simulation. At this point, 40 generations were generated to achieve a population size of 21,000 animals. Then, 20,000 females and 300 males from the last historical population were selected as founders, followed by 15 generations of selection for estimated breeding values from best linear unbiased predictions and random matings. During these generations, replacement ratio were set at 0.83 and 0.45 for males and females, respectively. A random sample of 2500 animals in generations 11 to 14 was used as training set, while the whole generation 15 was used as testing set (1500 animals). Phenotypes were simulated as a Gaussian distribution with heritability equal to 0.25. Then, the phenotype of the animals was coded as 0 or 1 depending on whether their simulated phenotype was below or above, respectively, of the population average (using only generation from 11 to 14), which creates a discrete scenario for the phenotypes.

A genome was simulated with 30 chromosomes 100 cM long. Two scenarios with different numbers of QTL were simulated. In the first, three QTL were randomly located along each chromosome with effects sampled from a gamma distribution. This generated 90 QTL affecting the trait that still segregated in the training population. A second scenario with 33 QTL per chromosome was also simulated with a total of 1000 QTL having some effect on the trait and following a traditional infinitesimal model specification.

Then, 9990 bi-allelic markers were uniformly distributed along the genome and coded as 0, 1 or 2, regarding the number of copies of the most frequent allele. Simulation was performed to obtain a linkage disequilibrium close to 0.33 (squared correlation of the alleles at two consecutive loci). Ten replicates were analyzed, and the mean and standard deviations are presented.

#### Discrete field set

A field data set was used here to illustrate the behavior of the methods in classification problems applied to genome-wide prediction of disease resistance in pigs. In this study we used one of the most important congenital diseases in pig industry as response variable: scrotal hernia (SH). Most affected individuals cannot feed effectively and consequently growth is affected [26]. This leads to higher feed costs, slower throughput, lack of product uniformity and consequent loss in income. In a nucleus breeding population, such individuals cannot be considered for use as breeding stock and effectively end up as culls. Heritability estimates around 0.30 and prevalence between 1% have been reported previously for this trait [27,28].

Data were provided by PIC North America, a Genus Plc company. The data set contained records of scrotal hernia incidence (score 0 or 1) in 2768 animals from three different lines. Animals from two purebred lines (A and B) were born in elite genetic nuclei, where environmental conditions were better controlled and risk of infections was lower. Animals from a crossbred line (C), from line A and other lines not used in this study, were born in commercial herds. Selection emphasis in line A was placed on reproduction and lean growth efficiency. Line B has been selected mainly for reproductive traits. Selection against scrotal hernia was equally emphasized in both lines A and B. The prevalence of the disease ranged between 1 and 2% in all lines. Genotypes of all animals with phenotypic records were obtained for 6742 SNP located in different genomic regions identified as candidate regions in previous studies [29,30]. A comprehensive scan under the available marker density was performed with all chromosomes being covered. After genotype editing following Ziegler et al. [31], 5302 SNP were retained, and all 923 total animals from line A, 919 from line B and 700 from line C were used. Fifty per cent of animals in the data set of each line were affected with scrotal hernia. For each individual and main effect for SNP *j*th, we defined two covariates $x_{j}^{1}$ and $x_{j}^{2}$, with $x_{j}^{1}=1$ if the genotype was aa (0, otherwise), and $x_{j}^{2}=1$ if the genotype was AA (0, otherwise).

Analyses within each line were performed leaving out the 15% youngest individuals, as testing set. The raw phenotype was used as dependent variable in a control case design. Note that systematic effects were not included as covariates for simplicity, although any covariate may be included in the algorithms without loss of generality. The predicted susceptibilities of animals in the testing set were the percentages of trees in a random forest that a given animal was considered as affected.

### Predictive ability

Performance of the models was based on predictive ability to correctly predict genetic susceptibility in the testing sets. The true genetic susceptibilities of individuals in the simulated data set are known. However, true genetic merits are unknown in the field data case. Therefore, predictive ability was evaluated in a different manner in the field data, as described below.

#### Simulated set

The true genetic susceptibilities were obtained from the simulations and followed a Gaussian distribution, whereas distributions of predicted susceptibilities were dependent on the model used. A Gaussian distribution was assumed for Bayesian regressions and an unknown distribution bounded between 0 and 1, representing the probability of individual *i *to be susceptible, for machine learning methods. Pearson's correlations were calculated between true and predicted genetic susceptibility merit for each model and simulated scenario.

In addition, the area (AUC) under the receiving operating characteristic curve was calculated for each model in each simulation. This curve is a graphical plot of the sensitivity, or true *vs*. false positive rate (1 − specificity) for a binary classifier system as its discrimination threshold changes [32]. The AUC can be used as a model comparison criterion and can be interpreted as the probability that a given classifier assigns a higher score to a positive example than to a negative one, when the positive and negative examples are randomly picked. Individuals with a true genetic susceptibility above or below the population average were assumed positive or negative cases, respectively. Models with higher values of AUC are desirable and are considered more robust.

#### Discrete field set

True genetic susceptibilities of individuals in the field data are unknown. Instead, estimated breeding values (EBV) for SH susceptibility obtained from routine genetic evaluation using the BLUP method [33] were assumed as the true genetic values. Routine evaluations included 6.9 million animals in the pedigree and approximately 2.3 million records of SH. The effects of line, litter, farm, and month of birth nested into farm were included in the threshold animal model used in the analyses. This may indeed be a crude approximation because EBV were calculated under a linear model with strong assumptions of linearity, additivity, non migration or non selection, although millions of records and animals are used in these genetic evaluations and the accuracy ranged between 0.50 and 0.96 for 95% of the EBV. To minimize the issue of this approximation, animals were classified as susceptible or non-susceptible. Non-susceptible animals were those in the lower *α *percentile of the EBV distribution in each line, whereas those in the upper (1-*α*) percentile were considered as susceptible (*α *∈ {5,10,25,50}). Lower values of *α *selected the more extreme animals, thus a smaller approximation error is expected.

Predicted accuracy was calculated between these EBV (**y**) and predictions ($\hat{y}$) in the testing set from methods TBA, BTL, RF, L2B or LhB. The predictive accuracy was estimated using misclassification rate, the phi coefficient correlation, sensitivity and specificity.

The phi coefficient correlation is the equivalent to the Pearson's product moment correlation for binary variables. It can be calculated as

$$r_{\varphi }=\frac{p(\hat{y}=1|y=1)-p(\hat{y}=1)p(y=1)}{\sqrt{p(\hat{y}=1)p(y=1)p(\hat{y}=0)p(y=0)}}$$

This coefficient may be not robust enough under certain circumstances such as those in which the categories are extremely uneven. Under these circumstances *r_ϕ _*has a maximum absolute value determined by the distribution of $\hat{y}$ and **y**.

Sensitivity and specificity for a given classifier may be computed as

$$\text{Sensitivity}=\frac{number\;of\;TN}{number\;of\;TN+number\;of\;FP},$$

and

$$\text{Specificity}=\frac{number\;of\;TP}{number\;of\;TP+number\;of\;FN}$$

Sensitivity measures the proportion of healthy animals that are identified as not being affected (TN = true negatives), whereas specificity measures the proportion of affected animals that are correctly identified as such (TP = true positives). Values of sensitivity and specificity closer to 1 are preferred. Specificity and sensitivity are more informative than raw rate of misclassification, as the latter does not differentiate if misclassification is on true healthy or true affected animals.

Furthermore, all animals in the respective testing sets were used to calculate the AUC statistic, described above, for each method within a line. Animals with SH were considered as positive examples, whereas animals without SH were considered negative examples. As stated before, AUC measures predictive ability and may be considered as a model comparison criterion. Higher AUC values are desirable, as mentioned above.

## Results and discussion

### Simulated data set

Table 1 shows the average predictive ability (standard deviations in parentheses) across replicates, measured as Pearson correlation, between true and predicted genomic values, and also using the AUC statistic for each model on each simulated data set. Machine-learning methods showed higher averaged accuracy in the simulated data set than Bayesian regression, although with a large standard deviation across replicates. Smaller differences between Bayesian regressions and machine-learning were found in the simulated scenario with 1000 QTL. TBA and L2B were the methods showing poorest accuracy (0.26 ± 0.10 and 0.24 ± 0.04, respectively) in the scenarios with 90 and 1000 QTL, respectively. The boosting algorithm, both L2B and LhB, achieved the highest averaged accuracy (0.37-0.41) in the simulated data set with a smaller number of QTL. In contrast, methods BTL and LhB showed better predictive ability in the 1000 QTL scenario, 0.35 ± 0.04 and 0.34 ± 0.06, respectively. Differences between methods within replicates were in accordance with the averages shown in Table 1, although standard deviations between methods across replicates were large. The AUC ranged between 0.61-0.66 for Bayesian regression and between 0.63 and 0.70 for machine-learning methods. Although similar values were found for all methods, RF showed higher and preferable classification performance according to this parameter (0.70 ± 0.07 for 90 QTL and 0.69 ± 0.04 for 1000 QTL). It is not possible to draw clear conclusions on the preferred method according to the number of QTL affecting the trait, in light of the results from the simulations. Nonetheless, there is a slightly better behavior of machine-learning on traits with a small number of genomic regions affecting the outcome of the trait. Previous studies have also shown good performance of boosting in dealing with different continuous traits in real data [8].

**Table 1:** Accuracy (standard error across replicates in parentheses), measured as Pearson correlation between predicted and true genomic assisted values, and area under the operating characteristic curve for different methods and number of QTL

	# QTL	TBA	BTL	RF	L_2_B	L_h_B
Pearson correlation	90	0.26 (0.03)	0.33 (0.04)	0.36 (0.04)	0.37 (0.07)	**0.41**^1 ^ (0.07)
	
	1000	0.32 (0.16)	**0.35 (0.01)**	0.30 (0.03)	0.24 (0.01)	0.34 (0.02)

AUC	90	0.61 (0.01)	0.65 (0.02)	**0.70** **(0.02)**	0.65 (0.04)	0.69 (0.03)
	1000	0.66 (0.01)	0.66 (0.00)	**0.69 (0.01)**	0.63 (0.01)	0.66 (0.01)

^1^Higher value is desirable; the best value is in bold face; TBA = Threshold Bayes A, BTL = Bayesian Threshold LASSO, RF = Random Forest; L_2_B = L_2_-boosting algorithm, L_h_B = L_h_-boosting algorithm

Bayesian regression showed larger Pearson correlations than ensemble algorithms in the scenario with a larger number of QTL. Method BTL achieved the largest Pearson correlation (0.38), followed by TBA and LhB (0.33). Method RF showed the smallest Pearson correlation (0.22) in this simulated scenario and the largest AUC (0.72). This suggests that RF ranked individuals less accurately than other methods when a large number of QTL affects additively the trait, but the method is more accurate than other methods at discerning between healthy and affected individuals.

It must be pointed out that the simulated scenarios are purely additive and other more realistic scenarios with a more complex interaction between genes and biological pathways might provide different results.

### Field data set

**Figure 1:**
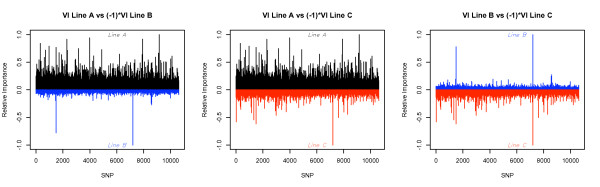
**SNP covariate relative variable importance (VI) in each line using random forest algorithm**.

The three data sets had a disease occurrence of 50%. The relative predictive importance obtained with RF for each SNP covariate $x_{j}^{l}$ in each line is plotted in Figure 1. Many more SNP were identified as predictors of SH in line A than in line B and C, suggesting that many more genomic regions may be associated to SH in line A than in line B or C. Lines B and C showed few genomic regions with a large relative importance variable associated to the genetic resistance to SH. Thirty seven, four and six SNP had a larger relative variable importance than 50% in lines A, B and C, respectively. The odds ratio of SNP with VI > 50% ranged from 1.41 to 2.17 in line A, from 2.56 to 3.03 in line B and from 1.86 to 2.50 in line C, suggesting a considerable risk of being susceptible to SH of those animals carrying the unfavorable alleles. The SNP with the largest importance estimate (VI = 100%) in line C had also the maximum VI in line B, but had a VI < 21% in line A. These results suggest that the genetic variants presented in line B and C in this genomic region provide a relatively larger predictive ability of SH than genetic variants in the same genomic region in line A. The relative VI of the most important SNP in line A was lower than 2% in lines B and C, although other SNP in LD with those may have been detected in these lines. Fifty, 44 and 48 markers with VI greater than 99.5 percentile were found in lines A, B and C, respectively. Most represented chromosomes were SSC4, SSC7, SSC14 and SSC17 in line A, SSC1, SSC2, SSC6 and X chromosome in line B, and SSC8 in line C. Validation of these results and conclusions about their role in genetic or biological pathways should be performed on different populations and studies.

Tables 2, 3 and 4 show the predictive accuracy of each method within lines A, B and C, respectively. RF had an equal or better predictive accuracy in the pure lines at α = 0.05, 0.25 and 0.50, than the rest of methods used in this study. Only L2B achieved a larger phi correlation (1.00) than RF (0.75) in line B at α = 0.05, and BTL showed higher accuracy at α = 0.10 in the purebred lines. Misclassification rate and sensitivity + specificity followed similar trends. RF and L2B were the most accurate at correctly detecting the most extreme animals in lines A and B, respectively, i.e. lower misclassification, and larger *r_ϕ_*, sensitivity and specificity were achieved at α = 0.05. RF and L2B achieved misclassification = 0, *r_ϕ _*= 1, sensitivity = 1 and specificity = 1 at α = 0.05 in lines A and B, respectively, which means a perfect classification of the most extreme animals. At this α level, TBA and BTL showed misclassification = 17%, *r_ϕ _*= 0.71 in line A and misclassification = 14%, *r_ϕ _*= 0.75 in line B, and were either less sensitive or specific than RF and L2B. RF outperformed BTL at α = 0.05, 0.25 and 0.50 in lines A and B, whereas TBL achieved better predictive accuracy at α = 0.10. RF doubled the *r_ϕ _*obtained with TBA at α = 0.50 in line A, and was 12% larger in Line B.

**Table 2:** Specificity, sensitivity, phi correlation and misclassification rate for each model at detecting different α and (1-α) percentiles of extreme animals in the testing set within line A

Parameter	Method	α (number of records)			
		**0.05 (12)**	**0.10 (79)**	**0.25 (98)**	**0.50 (138)**
		
Specificity^1^	TBA	**1**	0.71	0.58	0.56
	BTL	**1**	**0.94**	0.75	0.74
	RF	**1**	0.88	**0.78**	**0.79**
	L_2_B	0.75	0.71	0.64	0.65
	L_h_B	0.75	0.71	0.61	0.67

Sensitivity^1^	TBA	0.75	**0.58**	**0.58**	**0.56**
	BTL	0.75	0.53	0.53	0.47
	RF	**1**	0.52	0.52	0.46
	L_2_B	0.75	0.48	0.48	0.51
	L_h_B	0.50	0.45	0.45	0.42

Phi correlation^1^	TBA	0.71	0.24	0.16	0.13
	BTL	0.71	**0.39**	0.27	0.22
	RF	**1**	0.33	**0.29**	**0.26**
	L_2_B	0.48	0.16	0.12	0.17
	L_h_B	0.24	0.13	0.06	0.09

Misclassification rate (%)^2^	TBA	17	39	42	43
	BTL	17	**38**	**39**	40
	RF	**0**	41	**39**	**38**
	L_2_B	25	47	46	42
	L_h_B	42	49	49	46

^1^Higher value is desirable; the best value for each percentile is in bold face;

^2^Lower value is desirable; the best value for each percentile is in bold face;

TBA = Threshold Bayes A, BTL = Bayesian Threshold LASSO, RF = Random Forest; L_2_B = L_2_-boosting algorithm, L_h_B = L_h_-boosting algorithm

**Table 3:** Specificity, sensitivity, phi correlation and misclassification rate for each model at detecting different α and (1-α) percentiles of extreme animals in the testing set within line B

Parameter	Method	α (number of records)			
		**0.05 (7)**	**0.10 (25)**	**0.25 (78)**	**0.50 (137)**
		
Specificity^1^	TBA	0.75	**0.86**	**0.74**	**0.75**
	BTL	0.75	**0.86**	0.61	0.58
	RF	0.75	0.57	0.48	0.37
	L_2_B	**1**	0.71	0.57	0.48
	L_h_B	0.75	0.71	0.57	0.63

Sensitivity^1^	TBA	**1**	0.95	0.64	0.58
	BTL	**1**	**1**	0.75	0.75
	RF	**1**	**1**	**0.95**	**0.94**
	L_2_B	**1**	0.72	0.56	0.64
	L_h_B	0.67	0.78	0.73	0.69

Phi correlation^1^	TBA	0.75	0.80	0.34	0.34
	BTL	0.75	**0.90**	0.34	0.32
	RF	0.75	0.70	**0.50**	**0.38**
	L_2_B	**1**	0.40	0.12	0.12
	L_h_B	0.42	0.46	0.28	0.32

Misclassification rate (%)^2^	TBA	14	8	35	34
	BTL	14	**4**	29	32
	RF	14	12	**19**	**31**
	L_2_B	**0**	28	44	43
	L_h_B	29	24	32	36

^1^Higher value is desirable; the best value for each percentile is in bold face;

^2^Lower value is desirable; the best value for each percentile is in bold face;

TBA = Threshold Bayes A, BTL = Bayesian Threshold LASSO, RF = Random Forest; L_2_B = L_2_-boosting algorithm, L_h_B = L_h_-boosting algorithm

**Table 4:** Specificity, sensitivity, phi correlation and misclassification rate for each model at detecting different α and (1-α) percentiles of extreme animals in the testing set within line C

Parameter	Method	α (number of records)			
		**0.05 (7)**	**0.10 (24)**	**0.25 (80)**	**0.50 (104)**
		
Specificity^1^	TBA	**1**	0.50	0.64	0.71
	BL	0	0.25	0.61	0.71
	RF	**1**	0.75	0.75	0.71
	L_2_B	**1**	**1**	**0.96**	**0.98**
	L_h_B	**1**	**1**	0.82	0.69

Sensitivity^1^	TBA	0.33	0.30	**0.54**	**0.53**
	BL	**0.5**	0.30	0.44	0.43
	RF	0.33	**0.35**	0.52	0.51
	L_2_B	0.17	0.20	0.15	0.15
	L_h_B	0.33	0.20	0.46	0.45

Phi correlation^1^	TBA	**0.26**	-0.16	0.17	**0.24**
	BL	-0.35	-0.35	0.05	0.15
	RF	**0.26**	0.08	0.26	0.23
	L_2_B	0.17	**0.20**	0.17	**0.24**
	L_h_B	**0.26**	**0.20**	**0.28**	0.15

Misclassification rate (%)^2^	TBA	**57**	67	43	**38**
	BL	**57**	71	50	43
	RF	**57**	**58**	**40**	39
	L_2_B	71	67	56	44
	L_h_B	**57**	67	41	43

^1^Higher value is desirable; the best value for each percentile is in bold face;

^2^Lower value is desirable; the best value for each percentile is in bold face;

TBA = Threshold Bayes A, BTL = Bayesian Threshold LASSO, RF = Random Forest; L_2_B = L_2_-boosting algorithm, L_h_B = L_h_-boosting algorithm

None of the methods was clearly preferred in the crossbred (line C), where similar phi correlations were found for RF, TBA and boosting, with larger robustness for LhB at α < 0.50. No differences were found between RF, TBA and LhB to correctly detect most extreme animals in the crossbred line. The Huber loss function was more robust than the squared sum of residuals at analyzing binary traits, in accordance with its resemblance with the L_1 _loss function.

RF showed consistently larger AUC values than the other methods whichever line (Table 5), whereas a clear trend was not extracted from the AUC values of other methods. For instance, the boosting algorithms had larger AUC values (0.66-0.67) than Bayesian regression (0.62) in line C, but lower in line A (0.55-0.60 vs 0.64-0.65). This result also suggests that RF is less dependent on the choice of the threshold for classifying healthy and affected animals, providing larger stability to the classification.

**Table 5:** Area under the receiver operating characteristic curve^1 ^for each model and breed line in the field pig data

	TBA	BL	RF	L_2_B	L_h_B
Line A	0.64	0.65	**0.67**	0.55	0.60
Line B	0.70	0.69	**0.73**	0.60	0.72
Line C	0.62	0.62	**0.67**	**0.67**	0.66

TBA = Threshold Bayes A; BTL = Bayesian Threshold LASSO; RF = Random Forest; L_2_B = L_2_-boosting algorithm; L_h_B = L_h_-boosting algorithm

^1^Higher value is desirable; the best value for each line is in bold face

The true genetic architecture of SH is obviously unknown and no conclusions on its relationship with the performance of the different methods can be extracted. There was no clear relationship between the preferred method and the number of relevant genomic regions identified in each line (Figure 1). The choice of the model to be used in genome-wide prediction of traits like SH may depend on the interest of the breeder. For instance, detection of susceptible animals was done more accurately in line A using RF, whereas the Bayesian regressions were preferred in line B. Thus, a different method may be desired depending on the objective of the breeding program. The model with higher sensitivity would be preferred in a breeding program aiming at eradicating a given disease or trait. In a specifity+sensitivity scenario, RF was the best method at α = 0.05, 0.25 and 0.50, and it also showed the larger AUC values, regardless of the line.

Results showed that RF had the lowest risk, among methods used here, of misclassifying animals for low-medium heritability discrete traits in all lines, although all methods had considerable misclassification risks at α = 0.50. However, in a disease resistance genome-assisted prediction context, for instance, we are mainly interested in correctly detecting the most susceptible or resistant animals (lower α values), and RF seemed to perform slightly better than the Bayesian regressions to detect susceptibility to SH in this population, mainly in line A. Note that the threshold versions presented here incorporate *n *liability variables to be estimated in the model, increasing the parameterization of the models, and therefore hampering their predictive ability.

Results from the analyses of the crossbred line were not conclusive, as different behaviors between methods were found for different α values. This may be explained by the larger genetic heterogeneity expected in line C which may not be captured with only 5000 markers.

A small number of animals was used in the testing set and only punctual estimates are given here. This may be important at low α levels with a smaller number of records. Uncertainty about these estimates may be reported from their posterior densities [34] in the case of Bayesian methods and using bootstrap or cross-validation strategies in the case of this version of RF [11]. Uncertainties are not reported in this study because this data set aims at serving just as an example of three different models applied to discrete traits in a genome-assisted prediction context without overloading the discussion. Furthermore, the preferred model may be case-specific.

The misclassification rate and the logit function were also used as splitting criteria in RF but with poorer predictive ability (results not shown). Here, hyperparameters were set as fixed, although it is possible to assign them a prior distribution for their estimation [35]. Nonetheless, a minor improvement on predictive ability is expected if the ad-hoc choice of the parameters is within a sensible range of values.

## Conclusions

Two Bayesian regressions (TBA and BTL) and two machine-learning algorithms (RF and boosting) were proposed here to analyze discrete traits in a genome-wide prediction context. Machine-learning performed better than Bayesian regression with a small number of QTL with pure additive effects. RF seemed to outperform other methods in the field data sets, with better classification performance within and across data sets. It is an elegant method with an interesting predictive ability for studies on discrete traits using whole genome information. It is also easily interpretable as it is based on naïve decision rules. The boosting algorithms may achieve high predictive accuracy if a case-specific loss function is used, although it may be influenced by genetic architecture. Comparison between Bayesian regressions was dependent on the data set used, although the threshold version of the Bayesian LASSO seemed to be preferred to the threshold Bayes A.

RF and boosting do not need an inheritance specification model and may account for non-additive effects without increasing the number of covariates in the model or computing time. Results from this study showed some advantages in the use of machine learning to analyze discrete traits in genome-wide prediction, although model comparisons for specific case problems are encouraged.

## Competing interests

The authors declare that they have no competing interests.

## Authors' contributions

SF participated in the statistical analyses, discussion of results, got access and edited the data, coordinated the study and helped writing the manuscript. OGR participated in the statistical analyses and editing of the data, development of the software, discussion of the results and drafted the manuscript. Both authors read and approved the final manuscript and contributed equally to this study.

## References

[B1] Meuwissen THE Hayes BJ Goddard ME Prediction of total genetic value using genome-wide dense marker maps Genetics 2001 157 1819 1829 11290733 10.1093/genetics/157.4.1819 PMC1461589

[B2] Gianola D Perez-Enciso M Toro MA On marker assisted prediction of genetic value: beyond the ridge Genetics 2003 163 347 365 12586721 10.1093/genetics/163.1.347 PMC1462425

[B3] Gianola D Fernando RL Stella A Genomic-Assisted prediction of genetic value with semiparametric procedures Genetics 2006 173 1761 1776 10.1534/genetics.105.049510 16648593 PMC1526664

[B4] Wright S An analysis of variability in number of digits in an inbred strain of guinea pigs Genetics 1934 19 506 536 17246735 10.1093/genetics/19.6.506 PMC1208511

[B5] Gianola D Theory and analysis of threshold characters J Animal Sci 1982 54 1079 1096

[B6] Villanueva B Fernández J García-Cortés LA Varona L Daetwyler HD Toro MA Accuracy of genome-wide evaluation for disease resistance in aquaculture breeding programmes Proceedings of the 9th World congress on genetics applied to livestock production: 1-6 August 2010; Leipzig http://www.kongressband.de/wcgalp2010/assets/html/0325.htm

[B7] Szymczak S Biernacka JM Cordell HJ González-Recio O König IR Zhang H Sun YV Machine learning in genome-wide association studies Genet Epidemiol 2009 33 S51 S57 10.1002/gepi.20473 19924717

[B8] Gonzalez-Recio O Weigel KA Gianola D Naya H Rosa GJM L_2_-Boosting algorithm applied to high dimensional problems in genomic selection Genet Res 2010 92 227 237 10.1017/S0016672310000261 20667166

[B9] Long N Gianola D Rosa GJM Weigel KA Avendaño S Machine learning classification procedure for selecting SNPs in genomic selection: Application to early mortality in broilers J Animal Breed Genet 2007 124 377 389 10.1111/j.1439-0388.2007.00694.x 18076475

[B10] Long N Gianola D Rosa GJM Weigel KA Kranis A González-Recio O Radial basis function regression methods for predicting quantitative traits using SNP markers Genet Res 2010 92 209 225 10.1017/S0016672310000157 20667165

[B11] Breiman L Random forest Machine Learning 2001 45 5 32 10.1023/A:1010933404324

[B12] Friedman JH Greedy functions approximation: a gradient boosting machine Ann Stat 2001 29 1189 1232 10.1214/aos/1013203451

[B13] García-Magariños M López-de-Ullibarri I Cao R Salas A Evaluating the ability of tree-based methods and logistic regression for the detection of SNP-SNP interaction Ann Hum Genet 2009 73 360 369 19291098 10.1111/j.1469-1809.2009.00511.x

[B14] Sun YV Bielak LF Peyser PA Turner ST Sheedy II PF Boerwinkle E Kardia SL Application of machine learning algorithms to predict coronary artery calcification with a sibship-based design Genet Epidemiol 2008 32 350 360 10.1002/gepi.20309 18271057 PMC2828904

[B15] Tanner MA Wong WH The calculation of posterior distributions by data augmentation J Am Stat Assoc 1987 81 82 86

[B16] Park T Casella G The Bayesian Lasso J Am Stat Assoc 2008 103 681 686 10.1198/016214508000000337

[B17] de los Campos G Naya H Gianola D Crossa J Legarra A Manfredi E Weigel KA Cotes JM Predicting quantitative traits with regression models for dense molecular markers and pedigree Genetics 2009 182 375 385 10.1534/genetics.109.101501 19293140 PMC2674834

[B18] González-Recio O Lopez de Maturana E Vega T Broman K Engelman C Detecting SNP by SNP interactions in rheumatoid arthritis using a two step approach with Machine Learning and a Bayesian Threshold LASSO model BMC Proceedings 2009 3 S63 20018057 10.1186/1753-6561-3-s7-s63 PMC2795964

[B19] Tibshirani R Regression shrinkage and selection via the lasso J Royal Stat Soc B 1996 58 267 288

[B20] Hastie T Tibshirani R Friedman JH The elements of statistical learning. Data mining, inference and prediction 2009 New York, Springer

[B21] Freund Y Schapire RE Saitta L, Morgan Kaufmann Experiments with a new boosting algorithmproceeding of the Thirteen International conference on Machine Learning: 1996; San Francisco 1996 148 156

[B22] Breiman L Bagging predictors Machine Learning 1996 24 123 140

[B23] Goldstein BA Hubbard AE Cutler A Barcellos LF An application of random Forest to a genome-wide association data set: Methodological considerations & new findings BMC Genetics 2010 11 49 10.1186/1471-2156-11-49 20546594 PMC2896336

[B24] Tibshirani R Bias, variance, and prediction error for classification rules Technical Report 1996 Statistics Department, University of Toronto

[B25] Sargolzaei M Schenkel FS QMSIM: A large scale genome simulator for livestock Bioinformatics 2009 25 680 681 10.1093/bioinformatics/btp045 19176551

[B26] Straw B Bates R May G Anatomical abnormalities in a group of finishing pigs: prevalence and pig performance J Swine Health Prod 2009 17 28 31

[B27] Lingaas F Ronningen K Epidemiological and genetical studies in Norwegian pig herds. II. Overall disease incidence and seasonal variation Acta Vet Scand 1991 32 89 96 1950855 10.1186/BF03547000 PMC8127902

[B28] Vogt DW Ellersieck MR Heritability of susceptibility to scrotal herniation in swine Am J Vet Res 1990 51 1501 1503 2396801

[B29] Plastow G Sasaki S Yu T-P Deeb N Prall G Siggens K Wilson E Practical application of DNA markers for genetic improvement Proceedings of the twenty-eighth National Swine Improvement Federation meeting: 2003; Des Moines 2003 150 154

[B30] Hu ZL Dracheva S Jang W Maglott D Bastiaansen J Rothschild MF Reecy JM A QTL resource and comparison tool for pigs: PigQTLdb Mammalian Genome 2005 16 792 800 10.1007/s00335-005-0060-9 16261421

[B31] Ziegler A Konik IR Thompson JR Biostatistical Aspects of Genome-Wide Association Studies Biom J 2008 50 1 21 10.1002/bimj.200710398 18217698

[B32] Green DM Swets JM Signal detection theory and psychophysics 1966 New York: John Wiley and sons

[B33] Henderson CR Best linear unbiased estimation and prediction under a selection model Biometrics 1975 31 423 447 10.2307/2529430 1174616

[B34] Sorensen D Gianola D Likelihood, Bayesian and MCMC Methods in Quantitative Genetics 2002 New York: Springer Verlag

[B35] Yi N Xu S Bayesian LASSO for quantitative trait loci mapping Genetics 2008 179 1045 1055 10.1534/genetics.107.085589 18505874 PMC2429858

